# Manual development and efficacy of Mindful Living Group activities to promote trauma healing during the COVID-19 pandemic in China

**DOI:** 10.3389/fpubh.2023.1102473

**Published:** 2023-02-16

**Authors:** Ya-Nan Niu, Di Long

**Affiliations:** CAS Key Laboratory of Mental Health, Institute of Psychology, Beijing, China

**Keywords:** Mindful Living Group, COVID-19 pandemic, trauma healing, body-mind wisdom, Plum Village mindfulness practice, interbeing, the polyvagal theory, social engagement system

## Abstract

**Introduction:**

Disasters can be traumatic with a profound and lasting impact on individuals. During the COVID-19 pandemic, our team developed the Mindful Living Group (MLG) activities manual based on Eastern body-mind wisdom and Western trauma healing theory to provide psychological assistance for trauma healing.

**Methods:**

In this study, we introduce a framework developed for the 10-session MLG activities manual, which consists of three core modules. Thirty-one participants living all over the country who had experienced traumatic stress resulting from the COVID-19 pandemic received the MLG intervention. This single-arm intervention study offered psychological assistance during the pandemic. The MLG intervention included 10 weekly 2-h sessions held online. Participants completed the initial interview, pre-test, post-test, and 1-month follow-up interviews. The effectiveness of the MLG activities manual was evaluated using psychological measures, including Self-Rating Depression Scale, Self-Rating Anxiety Scale, Mindful Attention Awareness Scale, Post-traumatic Growth Inventory, General Self-Efficacy Scale, and the Perceived Social Support Scale.

**Results:**

Compared with the pretest level, the post-test levels of depression (*F* = 42.78, *p* < 0.001, *η*^2^ = 0.59) and anxiety (*F* = 23.40, *p* < 0.001, *η*^2^ = 0.44) were significantly lower; and mindfulness (F = 12.98, *p* =0.001, *η*^2^ =0.30), posttraumatic growth (*F* = 27.06, *p* < 0.001, *η*^2^ = 0.48), general self-efficacy (*F* = 13.20, *p* = 0.001, *η*^2^ = 0.31), and perceived social support (*F* = 16.27, *p* < 0.001, *η*^2^ = 0.35) were significantly higher (ANOVA). Further correlation analysis revealed a significant negative relationship of mindfulness with both depression (*r* = −0.43, *p* = 0.015) and anxiety (*r* = −0.35, *p* = 0.053), and significant positive relationships of mindfulness with posttraumatic growth (*r* = 0.40, *p* = 0.025), general self-efficacy (*r* = 0.52, *p* = 0.003), and perceived social support (*r* = 0.40, *p* = 0.024).

**Discussion:**

These preliminary findings showed the effectiveness of MLG activities for trauma healing. The mechanisms underlying mindfulness promoting trauma healing are discussed based on both Eastern body-mind wisdom and Western theories of trauma healing.

**Clinical trial registration:**

Identifier, ChiCTR2000034164.

## 1. Introduction

As an infectious pandemic, COVID-19 has had a profound and lasting traumatic impact on the global population. The prevalence of post-traumatic stress disorder (PTSD) has been estimated at 1–67% in some countries (1). Other psychological morbidities associated with the COVID-19 pandemic include poor sleep quality, stress, psychological distress, insomnia, anxiety, and depression (2). Poorer self-efficacy and social support (3) during the COVID-19 pandemic than before the pandemic have also been reported. Nevertheless, individuals can achieve positive experiences from adversity (4, 5), such as posttraumatic growth (PTG) (6). Identifying effective interventions and treatments will help individuals to heal trauma caused by disasters.

Clinical treatment for PTSD, including prolonged exposure therapy and cognitive processing therapy, focuses on trauma symptoms and has been shown to reduce PTSD symptoms (7–9). However, there is a high dropout rate during treatment (9, 10) and some patients continue to experience residual symptoms after treatment (8, 9). Moreover, treatments have often resulted in poor outcomes in patients with chronic or complex PTSD, such as PTSD combined with dissociation (11). Additional adjunctive or alternative treatments for trauma healing need to be explored (12).

It has been suggested that body-oriented treatments, rather than trauma-focused approaches, are more effective strategies (13, 14), such as somatic experiencing therapy (14, 15). Peter Levine proposed that trauma is retained in the nervous system of an individual's body, which is followed by the brain, such that talking therapies alone cannot offer sufficient healing. He suggested that individuals experiencing trauma should begin their healing process by learning to cultivate a perception of their body sensations and an awareness of their body's feelings (14). This is in agreement with the mindfulness practice that originated from the ancient Eastern tradition (15). As Zen Master Thich Nhat Hanh explained, “mindfulness is a kind of energy that we generate when we bring our mind back to our body and get in touch with what is going on in the present moment, within us and around us” (16). Emerging mindfulness-based interventions (MBIs) have shown effectiveness in the treatment of PTSD.

Among the mostly cited MBIs are mindfulness-based stress reduction and mindfulness-based cognitive therapy, which were initially developed for the rehabilitation of chronic illnesses (17) and to prevent relapse among patients with major depressive disorder (18), respectively. Both MBI types have been reported to effectively reduce PTSD symptoms such as re-experiencing, avoidance, emotional numbing, and hyperarousal (19–21) and to alleviate depression (19, 20, 22, 23), anxiety (20, 23), shame (22), and self-blame (21). Other MBIs, including loving-kindness meditation (24), mantrum repetition approaches (25), and transcendental meditation (26) have also been reported to have good efficacy in PTSD treatment.

The polyvagal theory, proposed by Porges, is one of the most important theories in trauma healing and has been applied in clinical therapies (27–31). According to the polyvagal theory, the evolutionary autonomic nervous system was composed of three subsystems: the dorsal vagal system (parasympathetic), the sympathetic system, and the ventral vagal system (also part of the parasympathetic nervous system). In individuals experiencing trauma, the sympathetic nervous system is strongly activated and the resulting constant state of fight-or-flight in response to unseen dangers leave them with fatigue, burnout, insomnia, and asthenia. In addition, the dorsal vagal nervous system can be activated, leading to frozen, collapse, and dissociation, which often occur in chronic or complex PTSD. Therefore, individuals experiencing trauma are often stuck in the past by traumatic memories and are unable to engage in the present moment (13). The activation or co-activation of the sympathetic and dorsal vagal nervous systems suppresses the social engagement system governed by the ventral vagal nervous system, making traumatized individuals isolated, which in turn aggravates their sense of powerlessness, helplessness, and hopelessness (13). The ventral vagal nervous system has evolved more recently and is unique to mammals; it acts to inhibit the excessive activation of the older dorsal vagal and sympathetic nervous systems and calms the body state (e.g., visceral homeostasis) to promote growth and restoration and to reduce stress through engaging socially with others. Most importantly, the social engagement system is integrated by bidirectional neural regulation between the visceral state and striated muscles of the face and head, which have an important function in social communication (32, 33). Therefore, this integrated social engagement system can be activated by both paths. One is to provide supportive environments to individuals with trauma through facial expression, eye contact, and voice to arouse feelings of safety and security. The other one is that individuals with trauma can also learn to calm their body and visceral state through body-mind practice to achieve inner peace and balance during daily life, in the absence of danger or threats.

In eastern tradition, mindfulness practices have been used for thousands of years to maintain a healthy body-mind state and to release oneself from suffering. Thich Nhat Hanh, who has brought mindfulness practices to the West since the 1970s, applied ancient wisdom to the challenges of modern life and developed Plum Village original teachings and practices of mindfulness for secular people. Plum Village mindfulness practice, also called “the Art of Mindful Living,” closely follows the Buddha's teachings and practices of mindfulness meditation, based on the Manifestation Only Psychology, with Mindfulness of Breathing (Anapanasati Sutta) and Four Establishments of Mindfulness (Satipatthana Sutta) as the guidelines. It provides a set of practical mindfulness activities to be practiced in all moments of daily life, such as mindful breathing, walking meditation, eating meditation, singing meditation, mindful movements, deep relaxation, deep listening, and loving speech (16, 34). These mindfulness practices are introduced in an explicit way to help people easily understand and enjoy their practice, which is expected to restore their inner peace and transform their sufferings. Moreover, Plum Village mindfulness emphasizes practicing within a group or community, which helps to rebuild connectedness with the self, others, and nature and helps to generate powerful collective mindfulness energy for healing. In Western trauma healing therapy, it has been suggested that a solidary and supportive group may provide the most direct, powerful, and persuasive experiences of connectedness and safety to people who have experienced trauma (35).

Our team have taken part in Plum Village retreats to practice mindfulness since 2007 and has applied these mindfulness practices to provide psychological assistance after the Wenchuan Earthquake disaster in China in 2008 to help people heal their trauma. We offered Mindful Living Group (MLG) activities to local people and practiced mindfulness with them over a period of more than 3 years and found that MLG activities were easily accepted and were effective in healing trauma. Since then, our team has continuously used MLG activities to serve different groups of individuals, such as those who experienced the Ludian earthquake disaster in 2014 and schoolteachers of primary and secondary schools in Beijing in 2017 and 2018, to restore body-mind energy, transform their suffering, and promote the healing process of trauma.

During the COVID-19 pandemic, our team programmed and developed the MLG activities manual based on Plum Village mindfulness practice combined with Western trauma healing theory, while removing the religious reference and expressions to provide psychological assistance to individuals experiencing trauma. This study evaluated the effectiveness of the intervention. To our knowledge, there are currently no published peer-reviewed studies that introduce a well-programmed mindfulness-based intervention based on Eastern tradition combined with Western trauma healing theory, and its effectiveness in an interventional study. Herein, we describe the development and effectiveness of MLG activities in promoting trauma healing during the 2020 COVID-19 pandemic in China.

## 2. Materials and methods

### 2.1. Participants

We recruited participants through the WeChat public account of the Institute of Psychology, Chinese Academy of Sciences across the country. Both recruitment and MLG activities were conducted online during the COVID-19 pandemic, allowing individuals living in different areas of China to participate. The eligibility criteria for enrollment in the study included individuals: (1) aged between 18 and 60 years; (2) experiencing mental distress during the COVID-19 pandemic, with symptoms such as insomnia, headache, chest tightness, fear, anxiety, guilt, self-blame, irritability, loneliness, and sadness; and (3) hoping to improve their body-mind states and return to enjoying their lives again, and (4) able to participate in online MLG activities with group members. Individuals requiring crisis intervention were not suitable for study participation and were instead referred to the appropriate services. Permission from clinical doctors was required for patients currently receiving medical treatment. The participants were preliminarily screened according to their registration information and further assessed during the initial interview. A total of 31 participants entered and completed the study. Demographic characteristics of the participants are presented in Table 2.

### 2.2. Procedures

#### 2.2.1. Development of the MLG activities manual

This study was funded by the Institute of Psychology, Chinese Academy of Sciences (No. E0CX331008, 2020–2021). We developed the MLG activities manual, drawing from Plum Village mindfulness practice and trauma healing theories, to provide psychological assistance during the COVID-19 pandemic.

The MLG activities manual consists of 10 sessions, including the first session of “Getting to know each other” and the final session of “Setting off again.” The eight themed sessions focus on three interconnected core modules, which are named “Watering our inner seeds of peace and joy,” “Transforming suffering,” and “Rebuilding connectedness” (see Table 1).

**Table 1:** The MLG manual framework of activities.

**Session**	**Theme**	**Aims**	**Mindfulness practices**
Session 1	1. Getting to know each other 2. Starting to live a mindful life	1. Introducing the MLG activities 2. Group members getting to know each other	• Mindful breathing • Deep relaxation
Module 1: Watering our inner seeds of peace and joy (sessions 2–4)	1. Experiencing the mindful life 2. Taking care of the body and mind, settled in the present moment	1. Learning basic mindfulness practices 2. Being aware of the mind-body state 3. Nourishing inner peace and joy 4. Accumulating mindfulness energy	• Singing meditation • Mindful breathing • Guided sitting meditation • Walking meditation • Eating meditation • Mindful Movements • Mindful reading • Mindful sharing
Module 2: Transforming suffering (sessions 5–7)	1. Embracing our suffering 2. Taking a deep look at our daily lives 3. Parents, myself	1. Looking deeply into daily life 2. Looking deeply into impermanence, non-self, and interbeing 3. Looking deeply into the “inner child,” having better understanding of one's parents	• Singing meditation • Guided sitting meditation • Deep relaxation • The Five Mindfulness Trainings • Mindful reading • Mindful sharing
Module 3: Rebuilding connectedness (sessions 8–9)	1. Touching the Earth 2. Connecting with the past, present, and future	Reconstructing life meaning by restoring a connectedness with the Earth Mother and past (ancestors and parents), present (self), and future (offspring)	• Singing meditation • Guided sitting meditation • Walking meditation • Touch the Earth • The Five Mindfulness Trainings • Mindful reading • Mindful sharing
Session 10	Setting off again	1. Reviewing the mindfulness practices 2. Looking forward on how to continue mindful living practices in the future	• Singing meditation • Guided sitting meditation • Mindful sharing and blessing

Following the first session, participants proceed to Module 1, “Watering our inner seeds of peace and joy,” which consists of three sessions. Participants engage in basic mindfulness practices together with 3–5 group leaders, including mindful breathing, sitting meditation, walking meditation, eating meditation, singing meditation, mindful movements, deep relaxation, deep listening, loving speech, and service meditation, all of which can be applied in daily life (16, 34). Singing meditation may help participants become settled with their bodies and surroundings with ease and joy and is practiced at the very beginning and at the end of each session. As sung in the mindful songs, “Happiness is here and now. I have dropped my worries. Nowhere to go, nothing to do, no longer in a hurry,” “Breathing in, I go back to the island within myself … Breathing out, I enjoy going back to my island,” and “The mind can go in a thousand directions. But on this lovely path, I walk in peace” (36). Mindful breathing and walking meditation are simple practices that can bring profound experiences to practitioners. By naturally following in- and out-breathing and becoming more aware of the contact of their feet with the ground, participants may come back to their body and fully live in the present moment, thus setting themselves free from regrets of the past and worries about the future. Sitting meditation may help participants feel relaxed, peaceful, and at ease in the present moment, thereby allowing them to become more concentrated. Sitting meditation and deep relaxation can be very healing and nourishing and can help participants restore their body-mind energy. Eating meditation can be helpful in achieving connectedness with food, other people, and nature, to experience the interbeing with the planet that is nourishing and sustaining us and can release feelings of loneliness and disconnection. Participants are encouraged to practice mindfulness during their daily routines and enjoy the moment.

It is expected that when practicing the basic mindfulness activities in Module 1, participants would become calm in their body-mind state, reduce unnecessary energy consumption, and nourish their inner peace and joy to inspire feelings of safety and security. Participants may also perceive peaceful facial expressions, voices, and smiles of others during mindfulness practice with other group members, in which, according to the polyvagal theory, the activated social engagement system and cultivated collective mindfulness energy contribute to heal each other.

Participants deepen their mindfulness practice in Module 2, denominated “transforming suffering,” which consists of three sessions. Each session focus on a specific theme that participants concern such as interpersonal relationship difficulties, distress within a family, dealing with bereavement and grief, and facing serious illness (e.g., cancer) and death. Focusing on each specific theme, guided sitting meditation (adapted from Plum Village practices) (37), the Five Mindfulness Trainings (16), mindful reading (selected short stories, poems, or essays about mindfulness practice), mindful sharing, and mindful responding (34) are conducted. Building on the energy of mindfulness and concentration accumulated during Module 1, Module 2 guides participants to practice looking deeply into what was happening within them and around them, which may bring insights such as Impermanence, Non-self, and Interbeing. These insights can help participants transform their suffering in a transcendent or sublimated way, which can help individuals with trauma to heal. However, it is difficult to look deeply without sufficient calmness and concentration (i.e., the practice of Stopping (止) in eastern tradition), which can be generated and strengthened by continuous mindfulness practice and the collective mindfulness energy created by all group members. Therefore, continuous group practice is required. Within the group, participants and group leaders can learn from each other's experiences and discoveries, and together will witness the wisdom of suffering transformation occurring in their lives.

Module 3, named “Rebuilding connectedness,” consists of two sessions, “Touching the Earth” and “Connecting the past, present, and future,” in which participants extend their practice of looking deeply. When touching the earth with their body, participants are guided to look deeply into the connectedness and continuity of ancestors (past), the self (present), their offspring (future), and all other creatures and materials (34). These practices can bring insight into the non-self in that personal suffering and inadequacy are shared human experiences, which can release participants from frustration, guilt, and self-criticism. Through this experience, participants can come back to the present moment to understand and integrate their trauma experience in an extended and continuous system (i.e., the whole nature of everything connected with each other) using Impermanence, Non-self, and Interbeing. The healing process occurs when participants can recount their life stories in an integrated way and are deeply listened to by other group members or when participants are enlightened by other group members sharing discoveries, rather than listening to a discourse by specialists. Touching the earth with their bodies also brings participants feelings of stability, security, warmth, and a sense of reality. In this way, participants can fully live in the present, knowing that past distress is not themselves, and can decide to live in the present mindfully, which is the only solid way to achieve a better future. In the final session, participants review the mindfulness practices together and discuss how to apply mindfulness practice in the future before setting off to continue their life journey. Table 1 shows the framework of the MLG activities manual.

Each MLG activity group consists of 10–20 participants with 3–5 group leaders. The group leaders work as a team and comprise counselors, social workers, and non-professionals with experienced mindful living practices. In this way, the intervention would deliver comprehensive life experiences and wisdom while avoiding professional authority over group members. Group leaders practice mindfulness together with others while guiding or responding to group members. Each participant in the MLG contributes their own practice to the collective mindfulness energy, regardless of whether they are group leaders or participants. When participants share their confusion or questions about mindfulness practice, group leaders respond mindfully, with both trauma-informed care knowledge and Eastern wisdom, such as Impermanence, Non-self, and Interbeing.

#### 2.2.2. Effectiveness evaluation of MLG activities

**Figure 1:**
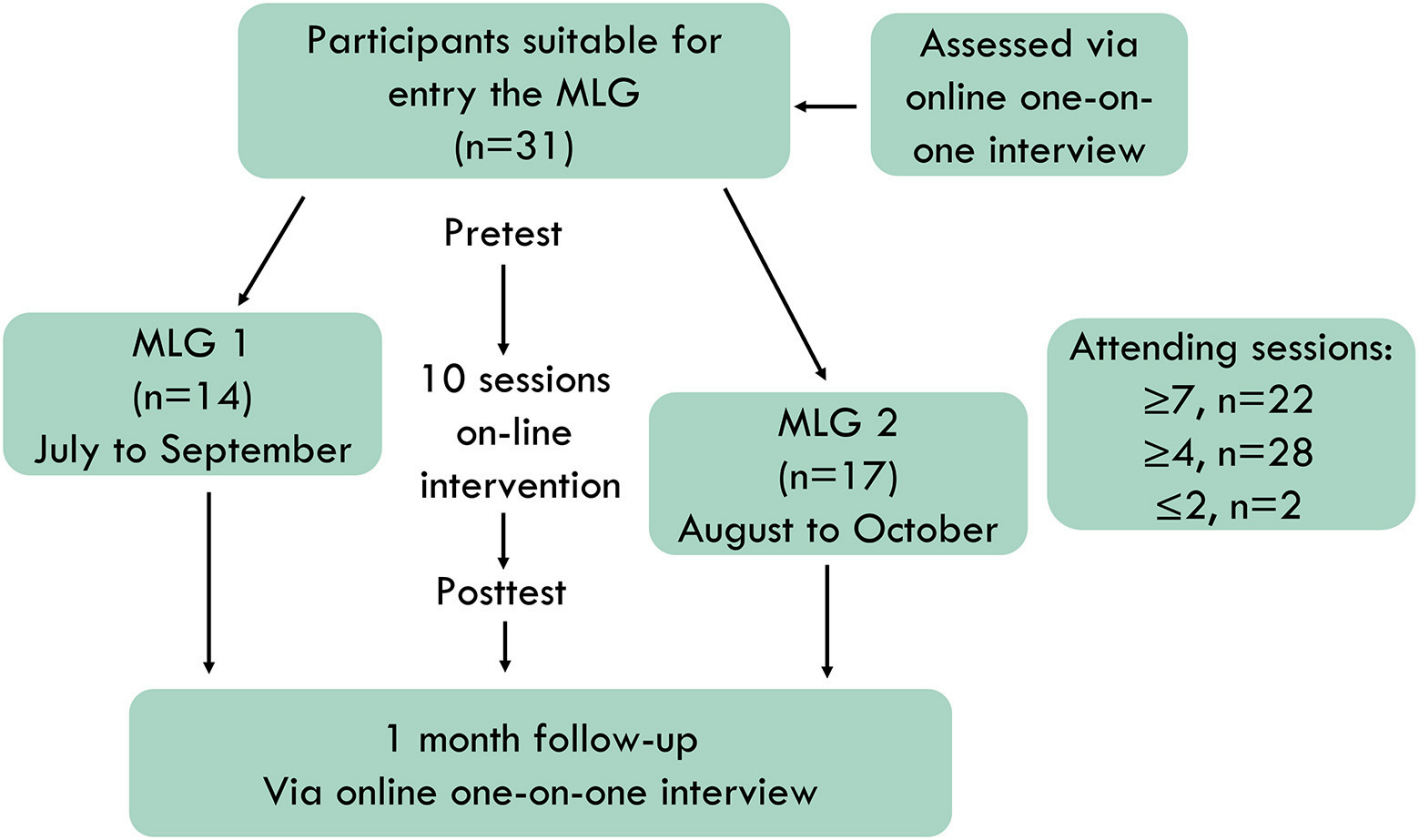
Flow of participants throughout the study.

Figure 1 shows the flow of participants throughout the course of the study. Participants' assessments for entry into the study were completed *via* an online one-on-one initial interview by the interviewers of our team. Written informed consent was obtained from all the participants. Data collection on demographic characteristics and outcome measures of the pre- and post-test periods was conducted using the Questionnaire Star platform (Changsha Ranxing Information Technology Co., Ltd). Follow-up was conducted 1 month after the MLG activities *via* online one-on-one interviews. Both online interviews and MLG activities were conducted *via* the Zhumu online meeting room (Suirui Technology Group Co., Ltd.).

Participants entered the MLG 1 or MLG 2 successively, according to their entry order and available time. The first MLG completed the intervention between July and September 2020. The second MLG completed the intervention between August and October 2020. Each intervention consisted of 10 weekly 2-h sessions. Of the 31 participants in both groups, 22 attended at least seven sessions, 28 attended at least four sessions, and two attended two or one sessions because of difficult working hours. Each participant completed the measures at both pre- and post-test, which were conducted within 1 week of the start and end of MLG activities. All participants completed the follow-up interviews. Data from all participants were included in the analysis.

### 2.3. Measures

The following measures were completed by participants in both the pre- and post-test periods.

#### 2.3.1. Self-rating depression scale

The Self-Rating Depression Scale (SDS) (38) was used to assess depression. The SDS is a self-reported scale used to measure the severity of depressive state and changes during treatment. The SDS consists of 20 questions that are scored on a Likert-type scale ranging from 1 to 4. The total scores range from 20 to 80, with higher scores reflecting more severe depression. The Chinese version of the SDS has been validated in various Chinese populations (39).

#### 2.3.2. Self-rating anxiety scale

The Self-Rating Anxiety Scale (SAS) (40) was used to assess anxiety. The SAS is a self-report scale used to measure subjective feelings of anxiety. The SAS consists of 20 questions that assess the frequency of anxiety symptoms within the previous week, which are scored on a four-point Likert-type scale. The total scores are summed and range from 20 to 80, with higher scores indicating more severe anxiety. The Chinese version of SAS has been validated and widely applied in clinical treatment and research (39).

#### 2.3.3. Mindful attention awareness scale

The Mindful Attention Awareness Scale [MAAS, (41)] is a 15-item self-report instrument used to measure an individual's mindfulness level. The frequency of mindful states over time was assessed, with higher scores indicating higher levels of mindfulness. Participants were asked to rate a series of statements on a six-point scale ranging from “almost always” (1) to “almost never” (6). The total score was obtained by summing the scores of all 15 items. The Chinese version of MAAS has been reported to have good reliability and validity (42, 43).

#### 2.3.4. Post-traumatic growth inventory

The Post-traumatic Growth Inventory (PTGI) (6) was used to measure the perceived benefits after a traumatic event. The questionnaire consists of 21 items covering five dimensions: relating to others, new possibilities, personal strength, spiritual change, and appreciation of life. Each item is scored on a 6-point Likert-type scale ranging from 0 (“no change”) to 5 (“a great deal of change”). Higher scores reflect greater perceived benefits from traumatic experiences. The Chinese version of the PTGI has been reported to have good reliability and validity (44). We added one item (“A better understanding of the existence of some uncontrollable force”) considering the cultural differences and interviews after the Wenchuan Earthquake of China (45).

#### 2.3.5. General self-efficacy scale

The General Self-Efficacy Scale (GSES) (46) was used to assess individuals' general perception of their ability to cope with difficult situations. The GSES is a 10-item self-report instrument and items are scored on a four-point Likert-type scale ranging from 1 (“not at all true”) to 4 (“completely true”). Higher scores indicate a higher level of self-efficacy. The Chinese version of the GSES has been reported to have good reliability and validity (47).

#### 2.3.6. Perceived social support scale

The Perceived Social Support Scale (PSSS) (48) was used to assess individuals' perceptions of the social support they received from family, friends, and others. The PSSS is a 12-item self-report instrument, and the items are scored on a seven-point Likert-type scale ranging from 1 (“very strongly disagree”) to 7 (“very strongly agree”). The total score is calculated by summing the scores of all 12 items, with higher scores indicating a higher level of perceived social support. The Chinese version of the scale has been reported to have good reliability and validity, including two dimensions consisting of social support within the family and outside of the family (39).

### 2.4. Data analysis

Data are presented as mean ± standard deviation (SD). Assumptions of a normal distribution were checked. A repeated measures one-way ANOVA was used to evaluate the effectiveness of participating in MLG activities on increasing mindfulness, reducing negative emotional symptoms, and promoting trauma healing. Pearson's correlation coefficient (r) was used to examine the relationships between the changes (i.e., pre-post-differences) in mindfulness and other measures, between the changes in general self-efficacy and other measures, and between the changes in perceived social support and other measures. Significance was set at 0.05 (2-tailed) for all analyses. All data analyses were performed using Statistical Package for the Social Sciences (SPSS version 22, IBM, NY, USA).

## 3. Results

The demographics of the two intervention groups are shown in Table 2. In addition to the traumatic experiences directly related to the COVID-19 pandemic, participants also talked about family trauma, bereavement, interpersonal relationship difficulties, income shock, vicarious trauma, sexual abuse in childhood, and serious illness (cancer). Some of the experiences occurred during the pandemic, whereas others were older traumas that predated the pandemic.

**Table 2:** Participants' demographic information (*N* = 31).

**Demographic features**	
Number of participants (group 1/group 2)	31 (14/17)
Mean age (SD)	37.06 (8.70)
18–29 years, *n* (%)	5 (16.13)
30–39 years, *n* (%)	15 (48.39)
40–49 years, *n* (%)	8 (25.81)
50–60 years, *n* (%)	3 (9.68)
Female, *n* (%)	27 (87.10)
History of mental illness diagnosis, *n* (%)	5 (16.13)
Religion of Buddhism, *n* (%)	2 (6.45)
**Level of education**	
Degree or professional qualification, *n* (%)	9 (29.03)
College or junior college qualification, *n* (%)	21 (67.74)
Senior high school or below, *n* (%)	1 (3.23)
**Traumatic experience**	
COVID-19 epidemic stress	18
Origin family trauma	9
Bereavement	8
Interpersonal relationship difficulty	8
Income shock	6
Vicarious trauma	4
Sexually abuse in childhood	1
Serious illness (cancer)	1
**Marital status**	
Single, *n* (%)	11 (35.48)
Married, *n* (%)	12 (38.71)
Divorced, *n* (%)	6 (19.35)
Widowed, *n* (%)	2 (6.45)
**Residence**	
Beijing, *n* (%)	8 (25.81)
Hubei, *n* (%)	7 (22.58)
Shandong, *n* (%)	4 (12.90)
Guangdong, *n* (%)	4 (12.90)
Jiangsu, *n* (%)	3 (9.68)
Hebei, *n* (%)	2 (6.45)
Shanxi, *n* (%)	1 (3.23)
Neimenggu, *n* (%)	1 (3.23)
Yunnan, *n* (%)	1 (3.23)

Table 3 shows the pre-post changes in the outcome measures. Compared with pretest scores, participants showed a significant decrease in SDS (*F* = 42.78, *p* < 0.001, *η*^2^ = 0.59) and SAS (*F* = 23.40, *p* < 0.001, *η*^2^ = 0.44) scores, which indicates that the level of depression and anxiety decreased after the MLG intervention. Compared with pretest scores, the average MAAS (*F* = 12.98, *p* = 0.001, *η*^2^ = 0.30), PTGI (*F* = 27.06, *p* < 0.001, *η*^2^ = 0.48), GSES (*F* = 13.20, *p* = 0.001, *η*^2^ = 0.31), and PSSS (*F* = 16.27, *p* < 0.001, *η*^2^ = 0.35) scores were significantly higher at the post-test assessment, indicating an increase in the level of mindfulness, post-traumatic growth, general self-efficacy, and perceived social support after the MLG intervention. Participants showed a significant increase in scores on all five PTGI dimensions at post-test vs. pre-test (PTGI-Personal Strength, *F* = 23.07, *p* < 0.001, *η*^2^ = 0.43; PTGI-New Possibilities, *F* = 16.84, *p* < 0.001, *η*^2^ = 0.36; PTGI-Relating To Others, *F* = 29.37, *p* < 0.001, *η*^2^ = 0.50; PTGI-Appreciation Of Life, *F* = 14.52, *p* = 0.001, *η*^2^ = 0.33; PTGI-Spiritual Change, *F* = 11.06, *p* < 0.01, *η*^2^ = 0.27; see Table 3). Participants showed significant increases in PSSS scores for both the within-family (*F* = 22.07, *p* < 0.001, *η*^2^ = 0.42) and outside-of-family (*F* = 10.69, *p* < 0.01, *η*^2^ = 0.26) dimensions.

**Table 3:** Scores on outcome measures pre- and post-MLG intervention (*N* = 31).

**Measure**	**Before mean (SD)**	**After mean (SD)**	***F*-value**	***p*-value**	***η* ^2^**
SDS	46.16 (7.03)	35.23 (7.68)	42.78	< 0.001	0.59
SAS	40.87 (8.34)	33.29 (6.52)	23.40	< 0.001	0.44
MAAS	55.32 (13.50)	62.32 (12.04)	12.98	0.001	0.30
PTGI	83.42 (22.1)	100.65 (20.36)	27.06	<0.001	0.48
PTGI-PS	15.06 (4.61)	18.61 (4.34)	23.07	<0.001	0.44
PTGI-NP	18.03 (6.19)	21.90 (5.62)	16.84	<0.001	0.36
PTGI-RTO	26.81 (6.88)	32.55 (5.91)	29.37	<0.001	0.50
PTGI-AOL	12.29 (3.63)	14.52 (2.58)	14.52	0.001	0.33
PTGI-SC	11.23 (4.22)	13.06 (3.71)	11.06	0.002	0.27
GSES	2.44 (0.48)	2.78 (0.50)	13.20	0.001	0.31
PSSS	53.16 (14.32)	63.29 (12.61)	16.27	<0.001	0.35
PSSS-WF	18.03 (5.55)	22.29 (4.63)	22.07	<0.001	0.42
PSSS-OOF	35.13 (9.82)	41.00 (8.91)	10.69	0.003	0.26

SDS, Self-rating depression scale; SAS, Self-rating anxiety scale; MAAS, Mindful attention awareness scale; PTGI, Post-traumatic growth inventory (–PS, personal strength; –NP, new possibilities; –RTO, relating to others; –AOL, appreciation of life; –SC, spiritual change); GSES, General self-efficacy scale; PSSS, Perceived social support scale; (–WF, perceived social support from within the family; –OOF, perceived social support outside of the family).

Further correlation analysis using the pre-post score difference revealed a significant negative relationship of mindfulness with both depression (*r* = −0.43, *p* = 0.015) and anxiety (*r* = −0.35, *p* = 0.053). Significant positive relationships were also found between mindfulness and posttraumatic growth (*r* = 0.40, *p* = 0.025), mindfulness and general self-efficacy (*r* = 0.52, *p* = 0.003), and mindfulness and perceived social support (*r* = 0.40, *p* = 0.024). When considering the PTGI dimensions, mindfulness was significantly associated with personal strength (*r* = 0.44, *p* = 0.014), relating to others (*r* = 0.38, *p* = 0.034), and appreciation of life (*r* = 0.51, *p* = 0.003). There were no significant correlations between mindfulness and new possibilities (*r* = 0.22, *p* = 0.226) or spiritual changes (*r* = 0.17, *p* = 0.375). When considering the PSSS dimensions, mindfulness was significantly correlated with perceived social support from people outside of the family (such as friends and colleagues) (*r* = 0.43, *p* = 0.015). Mindfulness was not significantly correlated with perceived social support from persons within the family members (*r* = 0.18, *p* = 0.344). Moreover, general self-efficacy was significantly correlated with decreased depression (*r* = −0.40, *p* = 0.026). The PSSS significantly correlated with the relating to others factor of the PTGI (*r* = 0.40, *p* = 0.025).

## 4. Discussion

Our team developed the MLG activities manual over a 10-year period, drawing from both Eastern mindfulness practice and Western trauma healing theory, and used this program to provide psychological assistance during the COVID-19 pandemic. The efficacy of interventions specified in the MLG activities manual was evaluated using psychological measures before and after the intervention combined with follow-up interviews.

The results showed that the MLG interventions may help cultivate mindfulness and decrease depression and anxiety. This is consistent with previous findings that MBIs, such as mindfulness-based stress reduction or mindfulness-based cognitive therapy, decreased depression and anxiety, and increased mindfulness (19, 20, 22, 23, 49). Recent systematic reviews and meta-analyses have demonstrated that MBIs that are based on mindfulness or use mindfulness as the foundation of therapeutic intervention, rather than integrating mindfulness elements or using mindfulness as an ancillary component (50, 51), have a moderate effect size on reducing depression and anxiety (51, 52). This demonstrates the efficacy of well-programmed MBIs in improving psychological wellbeing.

Our results also suggest that engaging in MLG activities can promote posttraumatic growth, general self-efficacy, and perceived social support, which could contribute to trauma healing. Correlation analysis further revealed that increased mindfulness was associated with decreased depression and anxiety and increased posttraumatic growth, general self-efficacy, and perceived social support. This indicates that the trauma-healing process may be promoted through increased mindfulness. Moreover, general self-efficacy was negatively associated with depression and perceived social support was positively associated with posttraumatic growth. Additionally, the analysis revealed that mindfulness is associated with increased personal strength, relating to others, appreciation of life, and perceived social support outside of the family. Together, these results demonstrate the efficacy of the MLG intervention in cultivating mindfulness to promote the trauma-healing process by improving general self-efficacy and perceived social support and reducing negative emotions. These results also support the use of the three modules of the MLG activities manual; that is, through mindfulness practice, participants can reduce unnecessary energy consumption, nourish their inner peace and joy, rebuild connectedness, and transform their suffering.

The present results are consistent with previous evidence of the efficacy of MBI in reducing PTSD symptoms (12) and promoting PTG (53). A recent meta-analysis reported significant associations between specific components of mindfulness and symptoms of PTSD, from the robust effect to the strong and small effects are the total mindfulness, acting with awareness, non-judging, describing, and non-reacting, and no significant association was found with observation (54). Another meta-analysis reported positive short-term effects of MBIs on PTG in participants with medical trauma (most were cancer-related) with a small effect size (53). Social support has also been reported to have a direct effect on PTSD and PTG, and affects PTSD negatively and PTG positively through self-efficacy (55, 56). Other studies have investigated the association between specific mindfulness traits and certain symptoms of PTSD (12, 54, 57). However, the mechanisms underlying the ability of mindfulness to promote healing of trauma remain unclear.

In this study, we referred to both Eastern body-mind wisdom and Western trauma healing theory. Body-mind unity has consistently been the theoretical and practical foundation for the traditional healing system of human beings; however, as Peter Levine stated, such an integration of body and mind has unfortunately been neglected in modern trauma treatment (58). Neuroscience studies have provided evidence for the existence of complex bidirectional interactions between the body and mind. For example, the polyvagal theory, which is the foundation for trauma healing therapy, proposes that perception of the inner body state (e.g., feeling peaceful or restless) influences the way people perceive their surroundings (e.g., as safe or dangerous) and vice versa (33). The social engagement system of individuals with trauma can only be activated to reduce stress when they perceive safety and security from both their inner body state and outer environment. When the body and mind are united in this manner, ventral vagal activities can be activated to suppress the excessive activities of sympathetic and dorsal vagal and promote body growth and restoration. In the Eastern tradition, mindfulness practices are used to achieve body-mind unity. For example, mindful breathing, which is the most commonly used practice, can directly influence the activity of the autonomic nervous system by decreasing the activity of the sympathetic nervous system and increasing that of the parasympathetic nervous system. This helps people feel relaxed and allows their mind to be present in their breath and body and live mindfully in the present. As another example, the embodied experiences of Impermanent, Non-self, and Interbeing, which can be achieved through practicing looking deeply based on body-mind unity or human-nature unity during mindfulness practice, brings individuals with a sense of connectedness and belonging and may also promote their social engagement system and the process of trauma healing. Further studies should be conducted to reveal the neural mechanisms underlying the effects of traditional practices.

For thousands of years, eastern traditional practices have provided systematic teachings and practice steps to follow. First, mindfulness (“nian,” 念 in Chinese) practice helps to bring our mind back to our breath and our body to the present moment, through which our body and mind are united. Second, continuous mindfulness practice and body-mind unity help to generate the energy of concentration (“ding,” 定 in Chinese), which means calm and still. Third, when our mind is calm and still, we are able to look deeply to understand and clarify what happens within and around us, through which insight (“hui,” 慧 in Chinese) can be attained, such as Impermanence, Non-self, and Interbeing. Transformation and healing can only take place when insight is attained. Mindfulness, concentration, and insight give birth to one another. The Plum Village method closely follows the eastern traditional teachings and practices, based on the Manifestation Only Psychology, with Mindfulness of Breathing (Anapanasati Sutta) and Four Establishments of Mindfulness (Satipatthana Sutta) as the guidelines of mindfulness meditation. In this study, the MLG activities manual is developed according to Plum Village teachings and practices of mindfulness. The three traditional practice steps (i.e., mindfulness, concentration, and insight) are involved in three interconnected modules (see Methods and Table 1).

Through the participants' sharing during the MLG activities and the follow-up interviews, we were able to understand how the embodied experiences with the insight of Impermanence, Non-self, and Interbeing promoted trauma healing over the 10 sessions and three modules of continuous mindfulness practice. For example, participant A with pandemic stress and vicarious traumatic experience attended all 10 sessions. After practicing eating meditation in the fourth session, despite other participants reporting feeling nourished and connected, she stated that she felt intensely uncomfortable when eating the food that she grew together with her neighbor, whose death in car accident had made it difficult for her to return to her vegetable garden. During the eighth session, after the guided meditation of looking deeply into connectedness with the past, present, and future, as well as with various elements in nature, such as the sunlight, water, air, earth, animal, plants, and minerals, the participant began to talk about the death of her nephew, which had caused her much distress for many years. She said that she realized during our group mindfulness practice that her nephew had gone his own way and she could start a life and enjoy her present life with her grandson. After sharing this with the group, she stated that she felt relieved, as if a huge stone had been expelled from her body. In the last session, “Setting off again,” she chose to attend the MLG activities while in her vegetable garden, showing us the vigorous grew vegetables in the sunlight, which inspired other group members and leaders.

Participant B shared the endless work-related stress experienced during the pandemic and the bodily feelings brought by mindfulness practice over the first 8 sessions. At the ninth session, she shared how, during the guided meditation, she was able to deeply examine her distress and understand that this was not a part of her and could be detached from her. At that moment, she felt hopeful for the future, because she knew that she could fully live in the present and thus make the future better.

Participant C attended six sessions due to work and family reasons and talked about her early trauma experience during the follow-up interview. “I thought I would bring my experiences to the grave without telling anyone else,” she said, “but I would like to talk about it now … I didn't share a lot during the MLG activities, and, more often, I sat there listening to others and watching the smiles on your faces. I was sure that I didn't need advice from anyone else because I knew what I should do. However, after these weeks attending the MLG activities, I found that I could talk about what I experienced.”

Participant D attended only four sessions due to work hour conflicts and talked about her difficult experience of providing assistance to hospitals during the early period of the pandemic. She was unexpectedly relieved to talk extensively about these experiences and feelings during the follow-up interview, as she was unable to speak about them before.

Participants started the MLG intervention with the common complaint of pandemic stress or related distress symptoms. However, each participant had a different requirement for healing given their different life experiences. It was not necessary for participants to share their traumatizing experiences in the past to accept the MLG intervention. In the MLG activities, participants decided individually when and how much to share of their mindfulness practice and the changes they were experiencing. Other group members practiced deep listening to the sharing. Together, the group members and group leaders witnessed changes and transformation within the group and learnt from and inspired each other. Most participants mentioned that it was important and meaningful for them to witness the peaceful state and smile of others, and the way that group leaders interacted with each other without worry or hurry helped them to relax. These findings agree with the polyvagal theory and suggest that social engagement can be achieved through group activities rather than through individual practice. In such grouped mindfulness practices, group leaders also reported being nourished and achieved growth together with the participants.

Although many MBIs are conducted in groups, they were more individual focused, lacking information on the interconnected nature of all lives, which is meaningful for trauma healing. As Deb Dana suggested, when people are anchored in ventral vagal regulation, they have a sense of being connected to others and the world. It is essential to help individuals experiencing trauma to create autonomic pathways of safety and connection (32, 33). The Plum Village method is based on the notion of Interbeing, a word coined by Thich Nhat Hanh, to help individuals to understand their deep connection to each other, nature, and all things. The insight of Impermanence, Non-self and Interbeing tells that everything changes constantly, “self” is composed of non-self elements because nothing exists independently and everything is interconnected and interacts with each other (59). If participants can perceive their interdependence and interconnection to others and the world in their daily lives, and behave with the awareness of interaction to each other, they are able to release themselves from sufferings and wrong perceptions, such as self-blaming, guilt, and shame caused by the senses of powerlessness, helplessness, and hopelessness. In fact, Impermanence, Non-self, and Interbeing are not religious terms exclusive to Buddhism, but these are universal concepts that exist across cultures. For example, the ancient Chinese philosophers Lao Zi and Zhuang Zi, who lived more than 2000 years ago, proposed that individuals should learn from nature, that is, they should understand that everything is interconnected and inter-transformed with each other in an endless succession. Individuals who adopt these perspectives in their lives can achieve release and ease. Therefore, we anticipate that Chinese individuals would be familiar with this discourse system and would be able to apply it wisely to heal themselves.

The body-mind healing wisdom also exists in Western therapies and is supported by scientific research. For example, Somatic Experiencing therapy, which is based on the polyvagal theory, helps individuals become aware of their inner body experience of interoception, kinesthesia, and proprioception by means of embodied imagery of kinesthesia and interoception; this helps clients to achieve biological completion of the thwarted response. Meanwhile, the therapist provides considerable social support using eye contact, verbal interaction, and all other factors that could draw clients toward a safe and comfortable state to activate their social engagement system and promote a balanced nervous system (15). Another recently reported body-mind treatment is the Moving to Emptiness Technique, which draws from core elements of traditional Chinese practice combined with western structural process. This technique helps clients remove their symptoms by means of transferring their target symptoms into perceivable objects and move them into a psychological emptiness realm through a series of embodied operations (60, 61). Comprehensive healing options can provide appropriate and personalized treatment that allow individuals to heal themselves and transform their suffering.

In this study, we did not observe any significant correlations of mindfulness with new possibilities or with spiritual change, which are two dimensions of posttraumatic growth. This might be a result of the limited 10-week period of the online MLG activities. Although this was not a short-term intervention as compared with other MBIs, new possibilities and spiritual changes might be generated from longer-term mindfulness practice. It might also be necessary for participants to have access to practical help, external resources, and services to generate new possibilities in their lives. The finding that mindfulness was not significantly associated with social support within the family suggests that family members should be involved in future MLG activities, for example, multifamily groups practicing MLG activities together.

In this study, we conducted MLG activities online due to the COVID-19 pandemic, which had both advantages and disadvantages. We recommend that MLG interventions be conducted in person to allow group members to interact with each other in a real-life setting, which favors the creation of real experiences and feelings that can generate a stronger collective energy of mindfulness than that obtained from remote online activities. However, conducting MLG activities over the internet is convenient and allowed people from all over the country to participate, with promising results. In addition, engaging in activities over the internet means that it is easy for participants to see their own expressions and the smiles on their own and on the faces of other participants.

## 5. Conclusion

In this study, we introduced the MLG activities that integrated the Plum Village original teachings and practices developed by the late renowned Buddhist Zen Master Thich Nhat Hanh and other trauma healing modalities developed by Western theorists and therapists such as the polyvagal theory proposed by Stephen Porges and Somatic Experiencing developed by Peter Levine. The effectiveness of interventions defined by the MLG activities manual was evaluated using psychological measures and was validated by the preliminary results of this study. We also discussed potential mechanisms underlying the promoting effect of MLG activities on trauma healing, while referring to both Eastern body-mind wisdom and Western trauma healing theory. Further studies using in-depth qualitative interviews are needed to further explore changes resulting from this intervention in the subjective experience of participants. In this study all participants received the intervention after enrollment, and there was no control or wait-list group due to the pandemic. Further studies with a more rigorous experimental design are needed, either off- or online, to examine the effectiveness of MLG activities in different populations.

## Data availability statement

The raw data supporting the conclusions of this article will be made available by the authors, without undue reservation.

## Ethics statement

The studies involving human participants were reviewed and approved by the Ethics Committee of the Institute of Psychology, Chinese Academy of Sciences. The patients/participants provided their written informed consent to participate in this study.

## Author contributions

DL designed the MLG program and the conceptualization of this article, and supervised the MLG activities and the manual development. Y-NN participated in the leading of MLG activities and drafted the manuscript. DL and Y-NN contributed to critical revision of the manuscript. All authors contributed to the article and approved the submitted version.
